# Refugee and Asylum Seeker Women’s Experiences with Healthcare and Social Environment in Malaysia

**DOI:** 10.3390/ijerph19116542

**Published:** 2022-05-27

**Authors:** Surendran Rajaratnam, Azlinda Azman

**Affiliations:** 1 Faculty of Social Sciences and Humanities, Universiti Kebangsaan Malaysia, Bangi 43600, Malaysia; 2 School of Social Sciences, Universiti Sains Malaysia, Gelugor 11800, Malaysia; azlindaa@usm.my

**Keywords:** refugee health, healthcare access, social work, Rohingya refugees

## Abstract

The internal conflict in Rakhine State, Myanmar over the last few decades has forced Rohingyas to flee to other countries, including Malaysia. However, the non-recognition of the status of refugees in Malaysia keeps Rohingyas as “people without documents” and without adequate protection, access to healthcare services, education, and employment. Women among these groups face different challenges and are at risk of numerous physical and mental health issues. Thus, this research attempted to understand the experiences of Rohingya women in Malaysia, particularly in accessing public hospitals. Focus group discussions and key-informant interview techniques were used to collect data. The transcripts were then analysed using the thematic analysis method. The research found that Rohingya women experience numerous challenges despite being on safer ground in Malaysia. Their experiences of marriage and domestic violence, access to public hospitals, financial barriers to healthcare services, and the services provided by medical social workers in the country to refugees and asylum seekers are presented. Non-recognition of the status of refugees in the country is one of the primary barriers to the allocation and provision of resources for refugees and asylum seekers. Due to structural barriers, medical social workers are unable to provide their services to this population. This article provides recommendations for social workers in Malaysia on how to overcome these challenges and work more effectively with refugees and asylum seekers.

## 1. Introduction

Rohingyas are a group of people from the Rakhine state of Myanmar who fled their country due to persecution and expulsion [1], to countries such as Bangladesh, Thailand and Malaysia. In fleeing their homeland, they lost their loved ones, their homes, and their possessions. They have a higher prevalence of communicable diseases including vaccine-preventable and water-borne diseases such as cholera, bloody diarrhoea, typhoid and hepatitis E [2,3] due to poor hygiene conditions in the camps and during their flight from conflict [4]. The population also suffers from a high prevalence of non-communicable diseases (NCD) such as hypertension and diabetes [3] which often goes undiagnosed or undermanaged. Additionally, the mental health of the refugees and asylum seekers is a concern because of the abuses and other forms of exploitations [3] and traumas they experienced. Studies have found mental health issues such as post-traumatic stress disorder (PTSD), depression and suicidal thoughts to be prevalent among Rohingyas [3,5].

Their difficulties do not end after fleeing their country. A new set of obstacles awaits them when they arrive in a new country. When they reach the shore or the boundary of a new country, they often realise that they may not be able to live the better life they had expected. Basic needs such as safe access to food, water, sanitation, shelter and healthcare services, may not be readily available to this population [6]. The physically and mentally agonising experiences associated with their forced migration and restricted or non-access to healthcare services in Malaysia contributes to the deterioration of their health [7] and potentially impacts the population of the host country. Rohingyas live in Malaysia as refugees and asylum seekers.

Refugees are people who have fled their own country because of a legitimate fear of persecution for various reasons, are outside their country, and are unable or unwilling to avail themselves of the protection of that country [8]; while asylum seekers are those whose claims for protection have not yet been decided by the country to which they have submitted it [9]. Refugees referred to in this article are those who possess a UNHCR registration card; while asylum seekers are those who are not documented or have their registration accepted by the UNHCR Malaysia, and do not possess the UNHCR registration card.

Although as refugees and asylum seekers, both Rohingya women and men have faced violence during the conflict in Myanmar, in-transit, and in Malaysia, their experiences of violence and its impact are different [10]. Rohingya women face difficulties with safety and security, sexual and reproductive health (SRH) issues such as safer pregnancy and delivery, marriage practices, gender-based violence (GBV) and other issues [11].

Most studies conducted to date on refugee women in Malaysia have explored issues from the public health perspective, which includes psychology and psychiatry [12,13,14,15] but not social work, despite its importance. Therefore, it is important to explore the challenges that Rohingya women refugees and asylum seekers face with respect to Malaysian healthcare services, and the role that social workers, within the context of healthcare and with other professionals, can potentially play in providing their services. The investigation will inform the development of policies and practices which align with the anti-oppressive principles of reducing their barriers to healthcare. This article reviews past studies to explore the barriers Rohingya women in Malaysia face in accessing healthcare services, and some of the reasons why Malaysian social workers cannot extend their services to the refugee and asylum-seeking population. It then briefly introduces the anti-oppressive social work theory as a backdrop to the arguments for social workers in Malaysia to provide their services to refugees and asylum seekers, which is put forth in this article. The research design, and methods used to conduct the research, are subsequently presented. To understand the physical and financial barriers to healthcare that Rohingya women face, and the services medical social workers provide in the country, the article presents primary data collected from refugees and asylum seekers, in addition to social workers and others who provided their services to these people. Building on these findings, the article offers a discussion on the physical, mental and financial challenges Rohingya women face, and medical social work services that are provided to refugees and asylum seekers in the country. Additionally, it offers recommendations and concludes with an argument for medical social work practice with this population in Malaysia.

## 2. Barriers to Healthcare That Rohingya Women Refugees and Asylum Seekers Face in Malaysia

In Malaysia, refugees and asylum seekers are not legally permitted to work and do not have access to free public healthcare or education. The country is not party to the 1951 Convention Relating to the Status of Refugees and its 1967 Protocol [16]. Therefore, a legal framework to distinguish a refugee or an asylum seeker from another undocumented migrant does not exist [17]. This leaves refugees, asylum seekers and stateless persons viewed as people without documents who can be fined, arrested, detained, imprisoned, punished and deported [17].

The challenges they encounter in Malaysia will lead them and their children to remain trapped in the cycle of poverty and other forms of disadvantages. The Rohingyas comprise the largest group of people among Malaysia’s registered refugee groups, followed by the Chins from Myanmar [18]. They are also one of the world’s most persecuted ethnic groups [19], whose people are highly vulnerable to various forms of deprivation. They are stateless because their citizenship in Myanmar has not been recognised, depriving them of social, political, educational, and healthcare rights [20].

To make matters worse, the lack of legal protection, the normalisation of violence within refugee groups, and the difficulty in accessing protection and justice for refugee women in Malaysia, contributes to an environment where they are particularly vulnerable to sexual and gender-based violence (SGBV) [21]. These challenges potentially lead them to life-threatening healthcare issues such as sexually transmitted diseases (STDs), unwanted pregnancies, menstrual disorders and psychological trauma [22]. These diseases often go undiagnosed due to many factors which include cultural barriers [23,24], financial constraints, and lack of access to healthcare services [24].

Although there are several non-governmental organizations (NGO)s and/or volunteer-run clinics for refugees in Malaysia [25], the high charges in hospitals and private clinics are the reason why refugees frequently visit refugee clinics. However, refugee clinics can only provide basic healthcare services; when refugees need advanced healthcare services, they will still need to be referred to hospitals.

Furthermore, undocumented women who seek healthcare assistance in hospitals face possible arrest in Malaysia. Health Equity Initiatives (HEI) reported incidences whereby asylum-seeking pregnant women who were admitted to a public hospital were told that upon delivery of their babies, they would be sent to a detention centre, which caused great anxiety to the women and their husbands on top of their usual fear associated with childbirth [13]. Fear of arrest, detention and deportation deterred these women from seeking professional healthcare assistance to deliver their babies, and may also lead to unsafe abortion or post-natal infection that risks maternal mortality [13].

## 3. Why Social Workers Could Not Provide Their Services to These Refugees and Asylum Seekers

NGOs and individual volunteers assist and support refugees in Malaysia to a certain extent. In the United States, for example, social workers engage and work with refugees from the time of the refugee’s arrival in the country. Each state in the country has its own social workers licensed to practice [26], and a board to regulate state licensing [27]. They provide basic needs for at least 30 days and perform case management and referrals to various social service providers. In New Zealand, the manager of the Refugee Resettlement Centre conducts an orientation programme, which includes language classes, health screening, and mental health support before resettling refugees into the country [28,29]. Similarly, in Western Australia, refugees go through post-arrival health screening, which includes trauma-informed and culturally appropriate healthcare services by a multidisciplinary team that includes social workers [30]. However, it must be noted that asylum seekers are predominantly smuggled into Malaysia [31], unlike those in the United States, New Zealand or Australia where UNHCR and other agencies located around the world assist in resettling refugees into the three countries from various other countries [32,33]. This enables the allocation and provision of systematic and adequate resources that includes social work services for asylum seekers or refugees.

In Malaysia, social workers are under the purview of the Department of Social Welfare within the Ministry of Women, Family and Community Development (MWFCD), while medical social workers are under the Ministry of Health (MoH). Medical social workers, of which the vast majority work in hospitals, perform administrative duties in addition to their duties to serve patients in need [34]. The roles these social workers play in the country are somewhat unclear because, inherently, social work is perceived to be almsgiving work, which does not require specialised training or qualification [35]. Other health professionals have been reported as performing some of the tasks of these social workers [35]. Due to the heavy workload, stressful nature of their jobs, inadequate workforce, and more importantly, a mandate, social workers are not able to provide even basic services such as preliminary assessment and referrals for asylum seekers and refugees who seek medical attention in public hospitals.

## 4. Anti-Oppressive Social Work

The anti-oppressive concept works on the premise of oppression. Oppression is defined as involving “relations of domination that divide people into dominant or superior groups and subordinate or inferior ones” [36]. The “superior” excludes the “inferior” from the available social resources through a systematic devaluation of the attributes and contributions of the latter [36]. Anti-oppressive social work was one of the approaches that fell under the umbrella of critical social work [37]. The social work profession, which is defined as one that “promotes social change, problem-solving in human relationships and the empowerment and liberation of people to enhance well-being”, strongly resonates the idea of anti-oppressive practice (AOP) [38]. Scholars have used and contributed to the anti-oppressive approach in both research and practice in the field of social work. At the micro-level category of practice, the limitations of anti-oppressive perspective in social work has been discussed and the use of critical consciousness was emphasized to fill its gaps [39]. On the other hand, an international comparative framework was used at the macro-level category of research to contextualise the debates and controversies surrounding AOP theorization in social work [38].

The anti-oppressive approach has also been utilised in various studies on refugees, including on the role social workers can play through their approach during the refugees’ initial settlement period in a host country. Two studies [40,41] explored an anti-oppressive practice model to support queer and trans refugees in Canada using critical intersectionality analysis. Another study [37] reflected on their practice of working with asylum seekers in Australia by delving into the experience of oppression and privilege in addition to a commitment to personal, cultural, and structural change.

Negative stereotypes among others is a manifestation of oppression that constrains the relations between the refugees and their host society as a whole [40]. Knowing the circumstances of the immigrants, and equipped with the information needed to counter stereotypes, social workers can engage civil societies to challenge the misrepresentation or distortion of facts inflicted upon this group of marginalised people [40].

## 5. Research Design

The research design employed to conduct this research was qualitative case study. Case studies are reported to have great value in building emergent knowledge in social work [42]. Primary data were obtained from the informants, who included Rohingya women refugees and asylum seekers, medical social workers, medical officers, volunteer workers/activists, refugee organization officers, and a mental health service provider (see Table 1). The Rohingya Women Development Network (RWDN), a Rohingya women NGO in Malaysia, collected data from Rohingya women informants. The organisation works with and/or for Rohingya women and girls in a wide variety of programmes which include advocacy, education, and women’s empowerment. This research used focus group discussions with two groups totalling 10 Rohingya women and in-depth interviews with another 12 Rohingya women. Key-informant interviews were conducted with four medical social workers and two medical officers in a public hospital in Malaysia. Other key-informants included two volunteer workers/activists, two refugee organisation officers, and one mental health service provider for refugees.

The age of Rohingya women informants ranged from 21 to 50 years. All, except for two, had at least one child. They were all born in Myanmar and had fled the country to Malaysia, except for one. Most (*n* = 18) fled Myanmar and arrived in Malaysia using Thailand as a transit country, while others (*n* = 4) used Bangladesh and Thailand. The FGDs took approximately one and a half hours to two hours, while the KIIs ranged from half an hour to one and a half hours per session.

The first author translated and transcribed interviews with Rohingyas together with Rohingya translators/transcribers who were proficient in both Rohingya and English languages. The authors recruited the translators/transcribers not only based on their language proficiency but also on their knowledge and years of experience of working with Rohingyas for several international and local organisations and the government. The translated and transcribed interview and focus group discussion transcripts were randomly selected to be re-assessed to ensure that the translation accurately represented the informant’s statements and their meaning.

This research used a thematic analysis method to organise and analyse the data collected. Thematic analysis is a method within qualitative data analysis that is conducted through identifying, analysing, and reporting patterns (themes) within data [43]. The researchers adapted and used the phases of thematic analysis [44] to perform data analysis for this research: (1) familiarize yourself with your data, (2) generate initial codes, (3) look for themes, (4) review themes, (5) define and name themes, and (6) write the thesis.

## 6. Ethics

All informants of this research were given an information sheet about the research. The informants then signed a consent form to participate in the research and allow the researchers to publish the information they provided without any of their identifying details (unless otherwise agreed). The informants were informed of their rights to refuse any questions or withdraw from the research at any point. Furthermore, the informants were advised that their participation in the research may not give them any direct benefits. A safe and comfortable space was provided to Rohingya women informants for the research. The researchers also employed a female Rohingya interviewer/facilitator and a note-taker, who were both well-versed in their language and had cultural sensitivity and awareness to lead the interviews and focus group discussions. The researchers compensated the Rohingya women’s time with raw basic food products, provided temporary child-care assistance while they were participating in the research, and transportation to the research location from their home. Informants of this research who had physical and/or mental health, or even other needs, were provided with assistance by RWDN and their network of relevant agencies or individuals (i.e., community leaders, activists).

## 7. Findings

The findings presented in the following four sections were selected based on the perspectives of the informants in this research. Only selected quotes and findings relevant to this article were chosen to be presented thematically here. Section 7.1 presents the experiences of women and girls being trafficked into the country and ending up in abusive marriages. Section 7.2 describes their experiences (including fears) and the experiences of pregnant women in accessing public hospitals. Section 7.3 reports on the financial barriers that Rohingyas face in accessing public hospitals, the influence UNHCR cards have on their accessibility, the sufferings they face, including mental health issues, and the difficulties in affording care. Section 7.4 reports on medical social work services, which are not provided to refugees and asylum seekers except for those under 18 years old.

### 7.1. Women and Girls’ Experiences of Marriage and Domestic Violence in Malaysia

Rohingya women and girls who fled from persecution and violence in Myanmar found themselves to be experiencing violence within their households in Malaysia. Out of all the refugees and asylum seekers in the country, Rohingyas had the most people facing domestic violence issues.

“*It (violence) is one of the highest, in terms of sexual, gender-based, most common is domestic violence. And in domestic violence, the ethnic group that is known to be having these issues are the Rohingyas*.”

(Refugee organization officer)

The informants of this research mentioned having witnessed other Rohingya women and girls experiencing violence and assault. Their experience of being raped, which resulted in them becoming pregnant, caused further issues in their marital life, especially when they were “married off” to Rohingya men without being informed of their pregnancy status. One of the informants reported having faced physical violence at the hands of her spouse.

“*I have witnessed with my own eyes that the women were raped very badly (before reaching Malaysia). After being in Malaysia, men buy those women. They didn’t know they are pregnant*. *When they know that they are pregnant, they start to argue with each other*.”

(Rohingya woman, 37 years old)

“*He abuses by words and says angrily, ‘I cannot move because I don’t have card, where can I go, how can I afford?’ Sometimes I was beaten violently that my body swelled*.”

(Rohingya woman, 25 years old)

The interviews we conducted further confirmed that these women and girls were trafficked into the country and married off to local Rohingya men regardless of their age. The issue of child marriage is difficult to address because it became a norm, and many of the community members hold on to it, especially the older men. Although the community is reported to practice child marriages, the extent to which it is prevalent in Malaysia is not known.

“*And there is also the issue of child marriages. There is still that practice among Rohingyas that if you are 14, 15, no more education for you…marry you off*.”

(Refugee organization officer)

Additionally, according to the refugee organization officer, the most common forms of SGBV faced by Rohingya women and girls in Malaysia were marital rape, domestic violence, child rape and sexual assaults. The perpetrators were usually people whom they knew such as their husbands, community members, locals, employers and teachers. Cases of SGBV by teachers at refugee schools were increasingly reported in the community. Additionally, the activist informant, who had worked with refugees for more than five years, reported that child marriages and the scale of sexual violence endured by Rohingya women and girls were appalling.

“*A few thousand girls between the ages of 11 and 15 or 16 that have been subjected to that kind of horrific sexual violence that you and I can’t even comprehend and then sold into marriages to Rohingya men, trapped forever in this institution, stupid institution called marriage, you know, having to endure sex at the whims and fancy of the husbands, the so-called ‘husbands*’.”

(Activist)

Consequently, the mental health problems of Rohingya women and girls were further affected by the violence they faced from their husbands and other family members.

“*when we ask, not all of them, but some, are willing to share more, have mental disorders before they come into Malaysia. We also have some patients who have already developed schizophrenia*”

(Mental health service provider)

### 7.2. Access to Public Hospitals for Refugees and Asylum Seekers

Rohingyas accessed public hospitals that were close to where they lived and worked. In the hospital where this research was conducted, a high number of Rohingya patients were seen because it was located near a wholesale market where a high number of people from this ethnic group live.

“*They work under the owners of the ’pasar borong’ (wholesale market), and they run the markets themselves. So, these ladies are the wives of those who are working there*.”

(Medical officer)

The registration and possession of a UNHCR card had a significant impact on Rohingyas’ access to healthcare services, particularly in public hospitals. Among Rohingya women and girls, those who are pregnant and those with serious health conditions were the ones most at risk, as they faced barriers to receiving medical assistance. The majority of the challenges and fears associated with pregnancy and delivery were shared by the younger Rohingya informants in this research. The process of registering refugees and asylum seekers for admission and treatment in hospitals was reported to be challenging. One of the volunteer informants of this research felt resistance from a hospital’s registration counter when she attempted to register one of the Rohingya women and children.

“*As soon as they know you are dealing with refugees, you get the same treatment the refugees being given*.”

(Volunteer)

Rohingya women, particularly those without a UNHCR card, reported they avoided visiting hospitals for fear of being arrested, detained or unable to receive treatment.

“*I heard if people don’t have UN cards, they go to hospital GH and if the people from the hospitals know that they don’t have cards, they called immigration and handed over them to immigration [officers] to arrest them*.”

(Rohingya woman, 18 years old)

Another informant reported that she knew a girl who was arrested and detained for five months in a detention centre after being discharged from hospital.

“*There are some people who were suffering from sickness, went to the hospital when they don’t have card or money, they depend on the contribution from the people, and they go to the hospital and after getting admitted in hospital and cured, police go there and arrest (them) and send them to the camp (detention centre*.”

(Rohingya woman, 25 years old)

Apart from the non-UNHCR card-holding Rohingyas facing barriers to accessing public hospitals, women among them specifically faced challenges in delivering their babies.

“*Since we don’t have (the UNHCR) cards, if we go to the general hospital to deliver the baby, the nurse will ask whether we have the card or not. If we cannot show them the card, they shout at us, and it is very difficult to make the book for the pregnancy and delivery*.”

(Rohingya woman, 23 years old)

Some Rohingyas also reported having fled halfway during treatment at the public hospital for fear of arrest and detention, in addition to fear of being asked to pay. An interviewed volunteer shared her experience of working with Rohingyas, who tended to flee due to their fear that the authorities will arrest and detain them for failing to pay their hospital bills.

“*And there are those that have successfully received healthcare assistance from the hospitals but what happens is that halfway through the procedures they run because they fear that they are unable to pay and the police will come after them. This happens in the Government hospitals. Many times, we have sort of raised funds, put them in, the procedures are half-way ongoing, and then they just remove all the machines and they just run*.”

(Volunteer)

### 7.3. Financial Barriers to Access Healthcare Services

The inability of refugees and asylum seekers to pay for their hospital bills and medicines has emerged as one of the main barriers to their access to public hospitals in Malaysia.

“*I think they always indicate that they have difficulties accessing care, and one of the main reasons is also because of affordability of care. Many of course work with very minimal pay, when they have a medical issue, they try to self-medicate. And then only go to the hospital or seek care only when they can’t take it anymore, when it gets serious. So most of them also have issue because of documentation*.”

(Refugee organization officer)

All Rohingya informants mentioned that they faced challenges accessing hospitals because they could not afford the cost of the treatment and medicine charged. The amount of money they earned was barely sufficient to run their family here in Malaysia and to remit some to their relatives in Myanmar. They said that when they were sick, they did not go to hospitals, but instead went to private clinics because it was more affordable.

“*With one person income, I have to pay for house rent, school for children, our food. So how can we go to the expensive hospital? Sometimes I go there when I can afford. Sometimes I have to send money to Akhyab (home), for my relatives. We don’t have a single cent as our savings. We are somehow just surviving*.”

(Rohingya woman, 37 years old)

The cost that they bore depended on whether they were registered with UNHCR and possessed its card. Refugees reported facing fewer issues with regard to admission and payment compared with asylum seekers (non-UNCHR card holders). Therefore, non-card holders were more likely to avoid public hospitals when they had healthcare issues requiring medical attention, than card holders.

“*If we go to hospital, it is very difficult to get treatment. We go to hospital when we don’t have any other way because of the [inability to earn/having little] income and house rent [that we have to pay], (informant implying that they don’t have any money left end of the month after paying for all the expenses to go to the hospital). If we go to the hospital, we are being charged too much money. We go to the hospital when we have no other way, since we don’t have UN card*.”

(Rohingya woman, 27 years old)

Rohingyas who were connected with any NGOs or Malaysian (local) individuals who could assist them with admission and could guarantee that their bills would be paid, could sometimes gain better access to a public hospital than the rest of the population.

“*Again, they will also be charged on the foreigner’s rate. No matter where they go, it will be the foreigner’s rate (higher charge)….And they can’t afford it. Due to this, most of them stopped going to seek (for) doctor’s assistance. A lot of them have died. And because they just can’t afford. I mean cancer patients, kidney failure, liver failure, they can’t get assistance, they just die. I don’t know how to put it, but they just die. I just know a lady’s husband who just died because he couldn’t afford*.”

(Volunteer)

Rohingya women faced challenges, especially when they delivered their babies, as the cost of childbirth in public hospitals was unaffordable for them. The cost increased if they needed any surgical procedure, suffered any complications, or if the baby needed further treatment and care.

“*And we have to save RM5000 to RM10,000 for surgery (caesarean section). If caesarean, we have to pay RM10,000 and if without caesarean, we have to pay RM5000. So feel very bad. Even (when) I am able to give birth to the children, I am not giving birth because of that because the charges are high*.”

(Rohingya woman, 45 years old)

The payment for childbirth for Rohingyas with UNHCR cards was less of an issue compared with those without the UNHCR cards.

“*If you come in labour, to pay as a foreigner is about, the deposit is about almost 3–4 thousand, so it is a lot of money*.”

(Medical officer)

The hospital’s billing department staff and nurses reported to ensure that the payment for delivering babies was completed before both the mother and baby were discharged from the hospital. Upon delivering her baby, one informant recalled that she was asked to pay her hospital bill before she was allowed to see her baby.

“*When they called my husband, they told him to bring the money. So, if we ask, ‘How much money do we need to pay?’, they ask 1000, 1500. If we can’t pay and if we want to see the baby, they say, ‘You can’t see the baby unless you can pay’*.”

(Rohingya woman, 33 years old)

Rohingya informants reported that they had been threatened to be handed over to the authorities if they failed to settle their hospital bills. A volunteer informant shared her experience of knowing Rohingya women who were unable to afford their delivery at public hospitals.

“*It is sad to know that you are expecting and you can’t do anything to keep a healthy baby, when you deliver, you can’t afford to have a delivery, so you opt to delivering at home and then when they do it at home, these aunties don’t know what they are doing, but they still do it, and then they get the mothers get infected, the child gets infected, and the child suffers, the mothers suffer. I’ve seen these things happen. It is actually quite depressing*.”

(Volunteer)

The affordability of obtaining medical treatment was also mentioned to be an issue for Rohingyas seeking treatment for mental illness. The high charges for consultation and medicines, in addition to the lower quantity of medicines given, required patients to visit doctors more frequently, which increased the drop-out rates of Rohingya patients with mental illness.

“*Yes, this is why usually they will default treatment. Especially for the non- documented, the non-UN card holders will need to pay RM120 for each consultation, and they are only given five days medication every time. For example, you know schizophrenia, these are lifelong disorder, they have to take the medicine no matter how. If you give them (medicines) for only five days, they might go for the second time, third time, they might not have the money to go for the fourth time. They will discontinue the treatment saying the symptoms is likely to be in control, and later you will see the patient relapse*.”

(Mental health service provider)

Apart from medical bills for themselves and their family members, Rohingya women informants also reported facing financial challenges affecting other aspects of their lives, as most were not earning an income. They undertook unpaid housework and depended on their husbands or other male family members for money most of the time. However, when any Rohingya patient faced challenges in paying their hospital fee, the community members reported that they banded together to help the patient.

“*Usually in their community, if one person, let’s say the lady who is pregnant, needs to go to hospital, they pool their funds. Yes, they pool their funds. So, sometimes they manage to pay. For cases they do not manage to pay, I have not come across any so far*.”

(Medical officer)

Although some may have received financial support, others did not because most people could not afford to provide such support. On the other hand, Rohingya women received social support from their extended family and friends. Social support relieved them from sadness, and provided them with needed information to navigate through life, in addition to companionship.

### 7.4. Medical Social Workers and Their Work with Refugees and Asylum Seekers

Similar to other public hospitals in the country, the services that medical social workers provided in the hospital where this research was conducted were for Malaysian citizens. They provided services to a range of patients with social issues such as victims of domestic violence, abused mothers, and children who were in need. They also provided services to those in need of practical assistance, which were not limited to financial support to receive treatment and purchase medical equipment, discharge planning, institutional placements and tracking of family members. As patients required referrals for social work services from medical officers from clinical departments, social workers only knew of patients who were referred to them. Furthermore, they only accepted cases and provided intervention to those who were eligible according to the department’s policy. The cases of patients in need of medical social work services were filtered for their eligibility to receive services, before in-depth evaluations were conducted.

“*If we were to follow our SOP (Standard Operating Procedure), we don’t take in foreigners, including refugees. If we get referrals, we will explain that it is not in our SOP to provide services for them. But we will advice on what they can do. If its billing, then they can settle with the billing department, but we can verbally tell them which NGO they can go to if they can’t settle*.”

(Medical social worker)

For practical assistance, which involves financial support and institutional placements, the medical social workers followed their SOP and rules set by the resource providers or donors.

“*As for non-citizens, for most of the cases, we will not provide any support for financial assistance and institutional placement. Because for financial support, we are bounded by agencies which provide those resources. They [resource providers] have made it clear that its only for citizens*.”

(Head of Department, Medical social worker)

Social workers reported that they frequently received calls from hospital wards for financial assistance for refugees and asylum seekers. One of the medical social workers related a case of a patient in need of financial support for psychiatric treatment, but the department was not able to provide any assistance for the patient.

“*There was a case where the doctor wants to do treatment for a psychiatric patient, the rate was quite high, so the doctor requested whether can go through JKSP (the Hospital’s Social Work Department)….they should have checked directly with HASIL (The Billing Department)*.”

(Medical social worker)

When medical social workers were unable to provide their services for the refugees and asylum seekers, they suggested that their clients approach other NGOs that might be able to assist them. These NGOs were suggested based on their willingness to provide financial support for medical treatments to non-Malaysians. The medical social worker stated that:

“*We can only provide advice to them, we can’t go in- depth. We can’t open [the patient case] file*.”

(Medical social worker)

However, under certain circumstances, medical social workers provided their services to non-Malaysian citizens. They handled cases involving children who were under the age of 18 years. This was because those who are below 18 are protected under the Child Act.

“*If they are underage, we have to accept them regardless whether they are refugee or foreigner*.”

(Medical social worker)

## 8. Discussion and Recommendation

This research explored the experiences of Rohingya women refugees and asylum seekers in accessing healthcare services, and the challenges social workers face in providing their services to these women. These women refugees and asylum seekers face numerous physical and mental health issues due to the challenges they faced in Myanmar, on their journey to, and while in, Malaysia [7,14,45,46]. Despite their needs, Rohingya women face barriers to accessing needed healthcare services in Malaysia due to insecure legal status and protection challenges related to security, livelihood, poverty, deprivation, and social exclusion [14]. Additionally, they also face social and cultural barriers [7].

The findings of this research add to the existing literature on the challenges Rohingya women face in Malaysia, and in accessing healthcare services in public hospitals. At the point of arrival in Malaysia, they have acute and immediate health needs due to the dangerous and difficult journey they endured and their predisposing illnesses [7]. Similar to past studies [10,11,47], these women reported violence and assault prior to their arrival and while living in Malaysia. This research found that their experience of domestic violence while in Malaysia could be linked to the trafficking of girls and women for marriage to Rohingya men in Malaysia. According to [48], married Rohingya girls have higher risk of gender-based violence (GBV), including IPV, and mental distress.

Environmental stressors, especially unemployment and money problems, are key triggers for the abusive behaviour exhibited by Rohingya men [48]. These victims of intimate partner abuse often do not seek for help from others due to fearing shame, social stigma, concerns about confidentiality, concerns about legal documentation and language barriers [46]. Apart from these factors, the acceptability and normalization of intimate partner violence by men among Rohingyas discourages help-seeking behaviour among the women, and those who do want to seek help prefer informal support such as from family and religious leaders [47].

While older Rohingya women’s attitudes are changing, married girls are still excluded from social and personal progress [48]. Involvement of married girls and women in a range of programmes which improve their social and personal functioning, such as IPV, mental health, and family planning, is highly pertinent for women’s empowerment [48,49]. There is a need for a comprehensive healthcare service [7] for refugees and asylum seekers beyond the basic services they can currently access in Malaysia.

This research found that the physical and mental healthcare needs of Rohingya women are not adequately being met due to multiple challenges that exist. Financial access to specialized healthcare services in public hospitals, especially during admission and treatment, is beyond the affordability range of Rohingya patients. Asylum seekers, more than Rohingya refugees, in this research, face a heavier burden of affording healthcare as they are not entitled to the refugee discount due to lack of the UNHCR card, similar to the findings by other studies conducted in Malaysia [7,14,50]. However, refugees also find the cost of healthcare services at government hospitals to be unaffordable despite the 50% discount on fees charged to non-citizens [14]. Rohingya asylum seekers and refugees are unable to afford healthcare services primarily due to their lack of legal status in Malaysia, which prevents them from engaging in formal employment [50].

These refugees and asylum seekers remain in low level and underpaid informal work [14] that generates low, insufficient income to afford living expenses, including healthcare services. They also face language barriers [51], which are worse for Rohingya women and girls who are often highly dependent on male family members and relatives [52]. While the UNHCR Malaysia provides payment for medical bills for some refugees, these are often difficult to access [53]. Similar to [7], this research found treatment and transportation costs influence refugees’ and asylum seekers’ access to healthcare services, especially those with chronic illnesses, who require longer term care and regular follow-up.

Medical social workers are unable to extend their services to refugees and asylum seekers in Malaysia due to several barriers. Currently, the SOP of social workers in Malaysian public hospitals explicitly lists the services that should be accorded to Malaysian citizens and non-citizens [54,55]. These structural barriers that link healthcare eligibility to the legal status of refugees and asylum seekers hinder medical social workers from delivering their services [56].

Previous studies conducted in another refugee-hosting, non-signatory country to the 1951 Refugee Convention and its 1967 Protocol, Bangladesh, similarly found Rohingyas face legal and administrative barriers to accessing health and security [57,58]. Given these challenges, there is a need for a change in policy and funding in addition to inter-sectoral coordination for social workers to provide their services to refugee and asylum seeker women [59]. Social workers need to undertake national level and state level policy advocacy to address the issues and needs of asylum seekers and refugees [56] generally, in addition to women and girls specifically. This macro-level practice of social work is crucial in refugee and asylum seeker host countries such as Malaysia, which lack legal recognition of this group of people’s rights.

Additionally, there is a need for social workers to identify, understand, and prevent oppressiveness through awareness of and overcoming the “issues of control that seek to justify bureaucratic aims rather than enhancing human well-being”, in line with anti-oppressive practice (AOP) [36]. If resources are in place to provide services for refugees, social workers need to understand the challenges that different groups of refugees and asylum seekers face, keeping in mind how their intersecting social identities, such as gender, age and religion affect their social functioning. They can also use the situation-focused approach to acquire a systemic understanding of the refugee situation in the country [60].

Apart from that, the position of refugee social workers, who have expertise in working with the refugee and asylum seeker population, needs to be created and placed in key government institutions where these groups of people frequent, such as hospitals, immigration departments, detention centres, and in areas or districts in the state where they are concentrated. Professionals with expertise and experience working with both the population and on gender issues need to provide capacity building, training, and/or mentorship for social workers.

In addition, given the complexities within the Rohingya community, refugee social workers need to work with boys and men to address the prevalent issues of domestic and intimate partner violence.

One of the limitations of this research is it did not go beyond gender binary in its analysis and reporting. The researchers did not explore the perspectives of men, non-binary Rohingya refugees and asylum seekers. Future research could focus on non-binary and transgender Rohingya refugees and asylum seekers, as there is a paucity of literature on these groups of people. The researchers also did not consider other intersectional social identities of Rohingya women, as it was beyond the scope of this research. Despite its limitations, the research provides an insight into the important challenges Rohingya women and girls face within their family, and in accessing healthcare services that they need.

## 9. Reflexivity and Positionality

In all social science research, a researcher’s positionality influences the way they conduct the research. Therefore, this section will be helpful in understanding our positionality and the lens we used for this research.

This research was conducted as part of the first author’s PhD project. The author has worked as a gender researcher in an organization focused on food systems, and has completed some training on the subject. The second author was the project supervisor, who is also a professor of social work with more than 20 years of experience in their field.

The first author initially had a brief engagement with Rohingya women refugees and asylum seekers in Selangor, Malaysia, through a volunteer worker, prior to designing and conducting this research. At the meeting, the experiences of the Rohingya community, namely, the challenges they faced in this country, were shared and further discussed. The author was in frequent communication with the volunteer and others, including a woman and a man from the community, who kept the researcher up-to-date on the assistance provided to and any new initiatives for Rohingyas. Most of the engagement was with women and girls, who were facing numerous challenges and lacked needed support.

The first author is aware of the privilege he holds (and bias he may have) as a man and having citizenship of a country, while the women and girls interviewed for this project are of a different gender and are stateless. The author was able to be conscious of his thoughts and actions through reflection moments during coffee sessions with the two ethnic Rohingya volunteers, who shared their personal experiences growing up and living their lives as refugees. Additionally, the project supervisor, as a female social work professor, ensured that the research was sensitive to women and girls from the Rohingya community.

## 10. Conclusions

The systematic discrimination, statelessness and targeted violence aimed at Rohingyas over the past few decades in Rakhine State, Myanmar, triggered hundreds of thousands of Rohingyas to flee their country for other countries, including Malaysia. The trauma and suffering they endured in their home country during the conflict, during the journey to, and in the host country, are physically, emotionally and mentally agonizing.

The protracted situation due to the non-recognition of their status in Malaysia further negatively impacts their state of being. Among this group of people, women and girl refugees are exposed to physical and mental health issues due to abuse and exploitation, including forced marriages. Therefore, this research attempted to understand the experiences and challenges that Rohingya women in Malaysia face with regard to their health, their experiences in accessing healthcare services, and the ways that medical social workers can enable this group of people to better access healthcare services in Malaysian public hospitals. The research found that Rohingya women were, and continue to face, numerous challenges which impinge on their physical and mental health. Despite their coping strategies, if policy makers, social workers, researchers and other stakeholders do not take measures at different institutional levels, Rohingya women in Malaysia will not be able to have better access to health, and consequently a better life.

## Figures and Tables

**Table 1:** Types and number of participants and interview location.

No.	Type of Tool and Participant	Location	No. of Groups/Participants	No. of Sessions
1	FGD with Rohingya women	Selangor	10 participants	2 sessions
2	KII with Rohingya women	Selangor	12 participants	12 sessions
3	KII with medical social workers	Selangor	4 participants/3 sessions	3 sessions
4	KII with medical officers	Selangor	2 participants/2 sessions	2 sessions
5	KII with volunteer worker/activist	Kuala Lumpur	2 participants/2 sessions	2 sessions
6	KII with refugee officers	Kuala Lumpur	2 participants/2 sessions	2 sessions
7	KII with mental health service provider	Kuala Lumpur	1 participant/1 session	1 session
**Total**			33 participants	24 sessions

KII—Key-informant interviews; FGD—Focus group discussions.

## Data Availability

The data presented in this study are available on request from the corresponding author. The data are not publicly available to retain informant privacy.

## References

[1] Wolf S.O. (2014). The Rohingyas Crisis: A Security Perspective from Bangladesh.

[2] Faruque A.S.G., Khan A.I., Nahar B., Islam S.M.R., Hossain M.N., Abdullah S.A., Khan S.H., Hossain M.S., Khan F.H., Prajapati M. (2021). Cholera outbreak in Forcibly Displaced Myanmar National (FDMN) from a small population segment in Cox’s Bazar, Bangladesh, 2019. PLoS Negl. Trop. Dis..

[3] Joarder T., Sutradhar I., Hasan M.I., Bulbul M.M.I. (2020). A Record Review on the Health Status of Rohingya Refugees in Bangladesh. Cureus.

[4] Banerjee S. (2019). The Rohingya Crisis: A Health Situation Analysis of Refugee Camps in Bangladesh.

[5] Riley A., Varner A., Ventevogel P., Hasan M.M.T., Welton-Mitchell C. (2017). Daily stressors, trauma exposure, and mental health among stateless Rohingya refugees in Bangladesh. Transcult. Psychiatry.

[6] Women’s Refugee Commission (2013). Top 10 Critical Needs Facing Refugees & Persons Displaced in Emergencies.

[7] Chuah F., Tan S.T., Yeo J., Legido-Quigley H. (2018). The health needs and access barriers among refugees and asylum-seekers in Malaysia: A qualitative study. Int. J. Equity Health.

[8] Douglas P., Cetron M., Spiegel P. (2019). Definitions matter: Migrants, immigrants, asylum seekers and refugees. J. Travel Med..

[9] Amnesty International (2019). What’s the Difference between a Refugee and an Asylum Seeker?. https://www.amnesty.org.au/refugee-and-anasylum-seeker-difference/.

[10] Akhter S., Kusakabe K. (2014). Gender-based Violence among documented Rohingya refugees in Bangladesh. Indian J. Gend. Stud..

[11] Yousuf R., Salam M.M., Akter S., Salam A. (2020). Safety and Security of Sexual reproductive Health and Gender-based Violence among Rohingya Refugee Women in Bangladesh. Int. J. Hum. Health Sci. (IJHHS).

[12] Wake C. (2014). Forced Migration, Urbanization and Health: Exploring Social Determinants of Health among Refugee Women in Malaysia. Ph.D. Thesis.

[13] Verghis S.E. (2015). Stop the Arrest and Detention of Asylum Seeking Women Accessing Maternal Health Care. http://www.healthequityinitiatives.com/stop-the-arrest-and-detention-ofasylum-seeking-women-accessing-maternal-health-care.

[14] Verghis S.E. (2013). Access of Chin and Rohingya Women Refugees and Asylum Seekers from Burma to Maternal Health Services in the Klang Valley, Malaysia. Ph.D. Thesis.

[15] Chuah F.L.H., Tan S.T., Yeo J., Legido-Quigley H. (2019). Health system responses to the health needs of refugees and asylum-seekers in Malaysia: A qualitative study. Int. J. Environ. Res. Public Health.

[16] Nah A.M. (2010). Refugees and space in urban areas in Malaysia. Forced Migr. Rev..

[17] Lego J.B.H. (2012). Protecting and assisting refugees and asylum-seekers in Malaysia: The role of the UNHCR, informal mechanisms, and the ‘Humanitarian exception’. J. Political Sci. Sociol..

[18] United Nations High Commissioner for Refugees. 2016. 498. https://www.unhcr.org/en-my/figures-at-a-glance-in-malaysia.html.

[19] Hamling A. (2015). Rohingya people: The most persecuted refugees in the world. Amnesty Int..

[20] Nordin R., Maliki D., Masrur D., Hashemi H. (2016). ASEAN Human Rights Dilemma: The Plight of the Rohingyas in Myanmar. Law Rev..

[21] Li Yuen J. (2021). Sexual and gender-based violence among refugee communities in Malaysia: A policy brief by Women’s Aid Organisation. https://wao.org.my/wp-content/uploads/2021/01/SGBV-Among-Refugee-Communities-in-Malaysia.pdf.

[22] United Nations High Commissioner for Refugees [UNHCR] (1995). Sexual Violence against Refugees: Guidelines on Prevention and Response.

[23] Refugee Council (2009). The vulnerable women’s project: Refugee and asylum-seeking women affected by rape or sexual violence. Lit. Rev..

[24] Victorian Foundation for Survivors of Torture (2005). Sexual Violence and Refugee Women from West and Central Africa.

[25] United Nations High Commissioner for Refugees (2009). Free Clinic in Kuala Lumpur Draws Nearly 700 Refugees.

[26] SocialWorkLicensure.org Social Work Licence Requirements by State. http://www.socialworklicensure.org/articles/social-work-license-requirements.html.

[27] Association of Social Work Boards Social Work Regulatory Boards & Colleges. https://www.datapathdesign.com/ASWB/LR/Prod/cgi-bin/LawBoardWebsiteDSWBDLL.dll/NewLAWBoards.

[28] McIntosh A., Cockburn-Wootten C. (2019). Refugee-focused service providers: Improving the welcome in New Zealand. Serv. Ind. J..

[29] Immigration New Zealand (2022). Mangere Refugee Resettlement Centre. https://www.immigration.govt.nz/about-us/what-we-do/our-strategies-and-projects/refugee-resettlement-strategy/rebuilding-the-mangere-refugee-resettlement-centre.

[30] Hirani K., Payne D.N., Mutch R., Cherian S. (2019). Medical needs of adolescent refugees resettling in Western Australia. Arch. Dis. Child..

[31] Ab Wahab A., Khairi A. (2020). Smuggling of Rohingyas from Myanmar to Malaysia: A Threat to Human Security (Penyeludupan Etnik Rohingya dari Myanmar ke Malaysia: Satu Ancaman Keselamatan Insan). Akademika.

[32] United Nations High Commissioner for Refugees [UNHCR] (2021). Information on UNHCR Resettlement. https://www.unhcr.org/information-on-unhcr-resettlement.html.

[33] Mitchell L., Kamenarac O. (2021). Refugee children and families’ positioning within resettlement and early childhood education policies in Aotearoa New Zealand. Kōtuitui N. Z. J. Soc. Sci. Online.

[34] Lim B.E. (2007). Study on the Job Satisfaction and Burnout among Medical Social Workers in Government Hospitals in Malaysia. Ph.D. Thesis.

[35] Crabtree S.A. (2005). Medical social work in Malaysia: Issues in practice. Int. Soc. Work..

[36] Dominelli L. (2002). Anti-Oppressive Social Work Theory and Practice.

[37] Nipperess S., Clark S. (2020). Anti-oppressive practice with people seeking asylum in Australia: Reflections from the field. Doing Critical Social Work.

[38] Rush M., Keenan M. (2014). The social politics of social work: Anti-oppressive social work dilemmas in 21st century welfare regimes. Br. J. Soc. Work..

[39] Sakamoto I., Pitner R.O. (2005). Use of critical consciousness in anti-oppressive social work practice: Disentangling power dynamics at personal and structural levels. Br. J. Soc. Work.

[40] Valtonen K. (2002). Social Work with Immigrants and Refugees: Developing a Participation-based Framework for Anti-Oppressive Practice Part 2. Br. J. Soc. Work.

[41] Lee E.O., Brotman S. (2013). Speak out! Structural intersectionality and anti-oppressive practice with LGBTQ refugees in Canada. Can. Soc. Work Rev..

[42] Greenwood D., Lowenthal D. (2005). Case study as a means of researching social work and improving practitioner education. J. Soc. Work Pract..

[43] Castleberry A., Nolen A. (2018). Thematic analysis of qualitative research data: Is it as easy as it sounds?. Curr. Pharm. Teach. Learn..

[44] Braun V., Clarke V. (2006). Using thematic analysis in psychology. Qual. Res. Psychol..

[45] Low S.K., Kok J.K., Lee W.Y. (2014). Perceived discrimination and psychological distress of Myanmar refugees in Malaysia. Int. J. Soc. Sci. Humanit..

[46] Tay A.K., Islam R., Riley A., Welton-Mitchell C., Duchesne B., Waters V., Varner A., Ventevogel P. (2018). Culture, Context and Mental Health of Rohingya Refugees: A Review for Staff in Mental Health and Psychosocial Support Programmes for Rohingya Refugees.

[47] Welton-Mitchell C., Bujang N.A., Hussin H., Husein S., Santoadi F., James L.E. (2019). Intimate partner abuse among Rohingya in Malaysia: Assessing stressors, mental health, social norms and help-seeking to inform interventions. Intervention.

[48] Guglielmi S., Mitu K., Seager J. (2021). ‘I Just Keep Quiet’: Addressing the Challenges of Married Rohingya Girls and Creating Opportunities for Change. Eur. J. Dev. Res..

[49] Tahir A.R.M., Adli S.A.S., Hashim R., Islahudin F.H. (2022). Reproductive Health Issues and Assessment of Knowledge, Attitude and Practice (KAP) on Family Planning (FP) among Rohingya Female Refugees. Open J. Soc. Sci..

[50] Wahab A.A. (2017). Rethinking refugees as economically isolated: The Rohingyas participation in informal economy in Klang Valley, Malaysia. J. ASEAN Stud..

[51] Nazri A.S., Talib K.A., Sulaiman N., Gidah M.E. (2022). Untangling the needs of refugees in Malaysia: The way forward. J. Nusant. Stud. (JONUS).

[52] Kojima Y. (2015). Rohingya Refugee and Migrant Women Shadowed by Sexual and Gender-Based Violence.

[53] Wake C., Cheung T. (2016). Livelihood Strategies of Rohingya Refugees in Malaysia “We Want to Live in Dignity”.

[54] Kementerian Kesihatan Malaysia (2018). Prosedur Operasi Standard: Pengurusan Bantuan Praktik.

[55] Kementerian Kesihatan Malaysia (2018). Prosedur Operasi Standard: Pengurusan Bantuan Terapi Sokongan.

[56] Potocky-Tripodi M. (2002). Best Practices for Social Work with Refugees and Immigrants. Best Practices for Social Work with Refugees and Immigrants.

[57] United Nations High Commissioner for Refugees (2007). Government of Bangladesh. Bangladesh: Analysis of Gaps in the Protection of Rohingya Refugees.

[58] Wali N., Chen W., Rawal L.B., Rawal L.B., Amanullah A.S.M., Renzaho A.M.N. (2018). Integrating human rights approaches into public health practices and policies to address health needs amongst Rohingya refugees in Bangladesh: A systematic review and meta-ethnographic analysis. Arch. Public Health.

[59] Wachter K., Cook Heffron L., Dalpe J. (2022). “We Weren’t Ready”: Provider Perspectives on Addressing Intimate Partner Violence among Refugees and Immigrants in The United States. J. Fam. Violence.

[60] Oduntan O., Ruthven I. (2020). Situational Information Behaviour. Int. J. Inf. Divers. Incl..

